# The Biological and Clinical Relevance of Inhibitor of Growth (ING) Genes in Non-Small Cell Lung Cancer

**DOI:** 10.3390/cancers11081118

**Published:** 2019-08-06

**Authors:** Elisabeth Smolle, Nicole Fink-Neuboeck, Joerg Lindenmann, Freyja Smolle-Juettner, Martin Pichler

**Affiliations:** 1 Division of Pulmonology, Department of Internal Medicine, Medical University of Graz, 8036 Graz, Austria; 2 Department of Thoracic Surgery, Medical University of Graz, 8036 Graz, Austria; 3 Research Unit for Non-Coding RNA and Genome Editing, Division of Oncology, Department of Internal Medicine, Medical University of Graz, 8036 Graz, Austria; 4 Department of Experimental Therapeutics, The University of Texas MD Anderson Cancer Center, UTHealth, Texas A&M College of Medicine, Houston, TX 77030, USA

**Keywords:** Inhibition of growth, ING, lung cancer, NSCLC

## Abstract

Carcinogenic mutations allow cells to escape governing mechanisms that commonly inhibit uncontrolled cell proliferation and maintain tightly regulated homeostasis between cell death and survival. Members of the inhibition of growth (ING) family act as tumor suppressors, governing cell cycle, apoptosis and cellular senescence. The molecular mechanism of action of *ING* genes, as well as their anchor points in pathways commonly linked to malignant transformation of cells, have been studied with respect to a variety of cancer specimens. This review of the current literature focuses specifically on the action mode of *ING* family members in lung cancer. We have summarized data from in vitro and in vivo studies, highlighting the effects of varying levels of *ING* expression in cancer cells. Based on the increasing insight into the function of these proteins, the use of *ING* family members as clinically useful biomarkers for lung cancer detection and prognosis will probably become routine in everyday clinical practice.

## 1. Introduction

In cells of healthy tissue, multiple mechanisms are necessary to control cell proliferation and maintain a balance between cell death and survival. Carcinogenic mutations in cancer genes allow cells to escape these governing mechanisms, permitting them to grow and proliferate indefinitely [1]. Within recent years, protein-coding and non-coding genes have been proposed as cancer drivers and tumor suppressors [2,3]. Tumor suppressor genes usually control growth-regulatory mechanisms, whilst genetic alterations affecting those tumor suppressor genes lead to metastatic spread [4,5,6]. Tumor protein p53 is one of the most frequently mutated tumor suppressors in human malignancies, however other genes exerting similar functions whose mutations also cause cellular control mechanisms to fail have recently been outlined: The inhibition of growth *(ING)* gene family is a powerful mediator of transcriptional regulation, regulation of the cell cycle, apoptosis, and cellular senescence [1]. All *ING* family members count among type II tumor suppressors since their inactivation leads to malignant transformation in different tissue types [7,8]. Until today, five human *ING* family genes *(ING1–5)* have been identified, with *ING1* being the most widely studied. Initially, *ING1* was isolated by means of subtractive hybridization between short segments of cDNAs [9]. The fragmented cDNAs were found to interfere with the activity of tumor suppressors by inhibiting protein synthesis through anti-sense sequences, or through truncated sense fragments that abrogate gene function in a dominant-negative fashion [9]. *ING2*, *ING3*, *ING4,* and *ING5* were outlined by homology search. *ING1* and *ING2* form a distinct subgroup, as they have been found to be evolutionally and functionally close [10]. Characteristic for the ING proteins is a high homology in their C-terminal domain, containing a nuclear localization sequence and a plant homeodomain featuring a high affinity to histone 3 tri-methylated on lysine 4 (H3K4Me3) [10]. H3K4-methylation has been reported to act primarily as a repressor of protein-coding genes, and this repressive function is ever-decreasing with age [11]. The methylation pattern of H3K4 has been linked to carcinogenesis, and may even have diagnostic and/or prognostic significance in numerous types of cancers [12]. All ING family proteins are involved in the control of cell growth, senescence, apoptosis, chromatin remodeling, and DNA repair. In recent years, *ING1* and *ING2* have been described as tumor suppressor genes in various human cancer types, and both *ING1* and *ING2* knockout mice were found to spontaneously develop malignant diseases such as B cell lymphomas and soft tissue sarcomas [13,14]. The highly conserved c-terminal plant homeodomain that is characteristic for the ING family, and is generally found in chromatin remodeling proteins, as well as the nuclear localization sequence and the nuclear conserved region directing the genes to the nucleus, allows the *ING* genes to interact with chromatin and possibly nucleosomes [15]. The N-termini differ between the *ING* members and define their specific functions, for instance, their antagonistic regulatory properties [16]. Moreover, it has been demonstrated that *ING* gene family members also exert their various functions on the epigenetic level [17]. They act as histone mark sensors, core components of histone deacetylases (HDACs) 1 and 2, and histone acetyltransferase (HAT) chromatin-modifying complexes. Thereby INGs affect the hallmarks of cancer by altering gene methylation patterns, acting mainly as tumor suppressors [17]. We have summarized the pre-existing data on *ING* family genes with their anchor points in non-small cell lung cancer in Table S1.

## 2. *ING1* and *ING2* Act as Tumor Suppressors in Human Lung Cancer

In an in-depth analysis of lung cancer tissue samples and cell lines, Okano et al. sought to identify whether *ING1* and *ING2* are aberrantly expressed in human lung cancer [18]. By means of PCR-single strand conformation polymorphism (PCR-SSCP) and DNA sequence analysis, the *ING2* gene was assessed. The expression of *ING1b* and *ING2* was analyzed using quantitative real-time reverse transcription (RT)-PCR. Thirty-one primary lung cancer tissue samples were paired to matched controls. The samples consisted of 14 adenocarcinomas (AC), eight squamous cell carcinomas (SCC), six small cell lung cancers (SCLC), two large cell carcinomas and one adenosquamous cell carcinoma.

In addition, the authors investigated 15 human non-small cell lung cancer (NSCLC) cell lines as well as 15 human SCLC cell lines [19]. In the cell lines, PCR-SSCP analysis was carried out, and each of the exons of *ING1b* and *ING2* was amplified by means of PCR primers. Afterward, DNA sequence analysis was performed [18]. The results of this analysis revealed aberrant expression patterns of *ING1b* and *ING2* in six out of 31 lung cancer tissue samples, and in only one out of 30 lung cancer cell lines, i.e., in the NSCLC cell line NCI-H23. According to the DNA sequence analysis, the aberrant bands revealed a G to A substitution at codon 173, which does not lead to amino acid substitution. When matched with healthy control tissues, the same mutation was found in these as well, suggesting that it is due to gene polymorphism [18]. T to C substitution at codon 13 was detected in mutated bands in exon 1 of *ING2*, which occurred in 6 out of 31 lung cancer tissues. Moreover, this T to C substitution does not alter the amino acid encoded, and the authors conclude that also this aberration was due to polymorphism. In the cell lines, mRNA of *ING1b* and/or *ING2* was up-regulated in seven out of eight lung cancer cell lines, as compared to the normal bronchial epithelium cell line BETA2A. Interestingly, all seven cell lines that exhibited up-regulation of *ING1b* mRNA also featured *p53* mutations, while the remaining cell lines expressed wild-type *p53* [18]. In seven out of eight lung cancer cell lines, *ING2* mRNA was down-regulated, and among six of the seven cell lines that featured *p53* mutation, reduced mRNA expression of *ING2* was observed as well. Summing up these findings, *ING1* and *ING2* are evidently expressed differentially in human lung cancer, as compared to healthy lung tissue. Thus, these genes may serve as prognostic disease biomarkers for lung cancer in the future [18].

In immunohistochemical analysis, Zhao and colleagues analyzed expression profiles of the ING2 protein in normal versus cancer tissues [20]. Among the cancer specimens tested, there were 192 samples of human lung cancer. In addition, mouse tissues were analyzed for ING2 expression, comprising, among others, bronchial and alveolar tissue samples. Both tissue types were tested positive for cytoplasmic as well as nuclear ING2 protein expression. Interestingly, in human tissues, ING2 was universally expressed in the cytoplasm while only certain tissues featured nuclear ING2 expression as well [20]. According to this study, ING2 expression in lung cancer occurred infrequently, as compared to breast, ovarian, and endometrial cancer where the majority of cases expressed ING2.

*ING1b* has also been investigated with respect to NSCLC carcinogenesis. Therefore, tissue samples from 88 NSCLC patients who had undergone surgery were studied [21]. The samples comprised 35 ACs, 48 SCCs and five samples of large cell carcinoma. PCR-SSCP sequencing was performed for *ING1* and the *p53* gene mutation, as well as quantitative RT-PCR for *ING1b*, *p21* and Bcl-2 associated X, apoptosis regulator *(Bax)* gene expression. For p21 and Bax protein expression, immunohistochemistry was performed. It was found that only two (2.3%) out of 88 NSCLC samples studied featured point mutations of the coding regions of *ING1b*. Standardized gene expression was determined by two pathologists who had no knowledge of the patients’ clinical data. In the 88 NSCLC samples studied, expression varied widely (0.768–0.404). In total, 37 carcinomas (42%) featured a diminished *ING1b* expression. No relation was found between *ING1b* expression and *p53* gene status [21]. With respect to *ING1b* gene expression, the standardized *p21* expression ratio tended to be higher in *ING1b*-positive tumors and was significantly lower in tumors that featured a reduced *ING1b* expression (*p* < 0.0029). Expression of the *Bax* gene also tended to be higher in *ING1b*-positive tumors and was significantly lower in tumor samples featuring a reduction in *ING1b* expression (*p* < 0.0001). The conclusion drawn from this analysis is an evident association of reduced *ING1b* gene expression with reduced *p21* and *Bax* gene expression in NSCLC [21]. This study was the first study to show this correlation. *ING1b* thus has a tumor-suppressive function in NSCLC. The mode of action is obviously connected to the expression of *p21* and *Bax*. However, the exact mechanism of this relationship still remains to be investigated in-depth.

In 2011, an interesting study was carried out by Luo and colleagues [22], investigating the role of *ING1* in lung carcinoma. Two lung cancer cell lines were transfected with recombinant *ING1b* plasmids. One cell line, A549, expressed wild-type *p53* whilst the other (SK-MES-1) featured *p53* mutation. Apoptotic rate, alterations in the cell cycle, cell growth rate and downstream p21waf1 protein expression were assessed in both transfected cell lines. Additionally, the p33ING1b-p53 complex was analyzed by means of co-immunoprecipitation. For the detection of gene aberrations and the expression pattern of *ING1*, 70 cases of fresh-frozen lung carcinomas and 217 cases of formalin-fixed paraffin-embedded (FFPE) lung cancer samples were examined for loss of heterozygosity (LOH), p33ING1b protein expression by PCR-SSCP, as well as immunohistochemistry. As a result, over-expression of *ING1b* was found to inhibit cell growth of both A549 and SK-MES-1 cells, induced cell cycle arrest and apoptosis. The p21waf1 protein was significantly up-regulated, and a complex of p33ING1b and wtp53 could be detected after the wtp53 lung cancer cells had been transfected by *ING1b.* LOH correlated inversely with the expression of p33ING1b, and p33ING1b expression was lost in the majority (53%) of lung cancer specimens overall [22]. The authors conclude *ING1* being a potent inhibitor of lung cancer cell growth, inducing cell cycle arrest and apoptosis by forming a complex with the wtp53 protein, and up-regulating p21waf1 [22].

*p33ING1*, *p53* and the autophagy-related gene *Beclin1* were investigated in NSCLC in another study from 2011, to find out more about the correlation between the expression of these genes with clinico-pathological parameters [23]. The researchers used NSCLC tissue gathered from operation, along with non-cancerous healthy lung tissue. mRNA, as well as protein expression of *p33ING1*, *p53* and *Beclin1,* were detected by means of RT-PCR and Western blotting. mRNA and protein expression of *p33ING1* and *Beclin1* in NSCLC were significantly lower as compared to the surrounding healthy lung tissue (*p* < 0.05). Expression levels of *p33ING1* mRNA and protein expression were found to be diminished in NSCLC specimens with good or middle differentiation, whilst they were higher in poorly differentiated NSCLC samples [23]. Conversely, the expression levels of *p33ING1* and *Beclin1* were lower in the presence of lymph node metastasis and higher with no lymph node metastases. Both markers were also expressed at higher levels in patients with stage I-II disease as compared to stage III-IV disease (*p* < 0.05). Interestingly, protein expression of mutated *p53* in NSCLC was significantly higher in the lung cancer tissues than in the surrounding non-cancerous tissues [23]. On the basis of this data, it is not conclusive whether *p33ING1* and *Beclin1* act as tumor-suppressors or as tumor-promoters in NSCLC. Nevertheless, the combined detection of *p33ING1*, *p53,* and *Beclin1* genes is suggested by the authors as a potential diagnostic tool for early diagnosis and estimation of prognosis in NSCLC [23].

Furthermore, ING1 has recently been investigated in the context of micro RNAs (miRNAs) by Jinag et al. [24]. MiRNAs have repeatedly been shown to be involved in NSCLC carcinogenesis, acting either as tumor promoters, or as tumor suppressors. In this study, miR-500 and miR-628 expression profiles were assessed by quantitative RT-PCR. Migration, invasion, proliferation, cell adhesion, and apoptotic rate were analyzed in order to gain knowledge about the function of these micro RNAs in NSCLC. Luciferase reporter assay was used to validate the direct targeting of *ING1* by miR-500 as well as miR-628 [24]. In NSCLC tissues, miR-500 and miR-628 were expressed at higher levels in comparison to noncancerous tissues. Inhibition of miR-500 and miR-628 led to significant suppression of NSCLC cell proliferation, migration, invasion, and cell adhesion, and induced NSCLC cell apoptosis. In addition, it was shown that the *ING1* gene was a direct target both for miR-500 and miR-628. *ING1* over-expression inhibited NSCLC cell proliferation, migration, and invasion, and made cancer cells undergo apoptosis, according to this research study [24]. Conclusively, miR-500 and miR-628 act pro-carcinogenic in NSCLC, both targeting the tumor suppressor *ING1.*

An immunohistochemical study by Pan et al. was conducted in order to analyze the role of ING2 in lung carcinogenesis [25]. Sixty-four samples of NSCLC were tested for ING2 protein expression by means of immunohistochemistry, and the results were confirmed by Western blotting. Additionally, RT-PCR was used to evaluate *ING2* mRNA levels as well. ING2 protein expression was found to be significantly decreased in this cohort of Chinese NSCLC patients [25], when compared to samples of normal lung tissue. ING2 was more frequently lost in AC (45.8%) than in SCC (26.3%). In the lung cancer samples, there was a shift of ING2 expression from the nucleus to the cytoplasm. Moreover, this study revealed a significant association between ING2 expression, lymph node metastasis, and TNM stage, albeit only for SCC and not for AC. According to this research study, ING2 is aberrantly expressed in NSCLC and is likely to contribute to lung carcinogenesis [25]. Down-regulation of ING2 protein expression was also confirmed by immunohistochemical analysis on 120 NSCLC specimens [26]. In >50% of the investigated samples, ING2 was down-regulated, which was more frequent in AC (68%) as compared to SCC (45%). No association with the patients’ gender, age, or five-years survival times were observed with respect to ING2. According to this study, no LOH or ING2 gene mutations were observed [26]. Notably, in 95% of the cancer samples examined, a silent single nucleotide polymorphism (SNP) was found. The authors of this study also investigated the promoter region of the *ING2* gene, however, no alterations in the methylation pattern were identified. Conversely to previous reports, no correlation between *p53* activity and *ING2* expression levels was seen in this study [26]. As a conclusion, also this data suggests an impact of *ING2* on NSCLC development.

Finally, it is also worth mentioning that *ING2* is the major target of the HDAC inhibitor vorinostat [27], which was proven efficient also in the treatment of NSCLC patients [28]. Interestingly, ING1 protein phosphorylation status impacts the cross-talk of the p33(ING1b) splicing isoform of ING1 with members of the 14-3-3 protein family [29]. The 14-3-3 proteins are expressed in human tissues universally and have the ability to interact with diverse signaling pathways, altering protein expression, function of kinases, phosphatases, and transmembrane receptors. Further, 14-3-3 binding resulted in a significant transfer of p33(ING1b) protein to the cytoplasm [29]. Hence, it is likely that 14-3-3 influences the activity of p33(ING1b) significantly by directing its subcellular localization [29]. A novel function of *ING2* was recently identified, i.e., the control of DNA replication and the maintenance of genome stability [30]. In small interfering RNA (siRNA) *ING2* cells, global replication rate was significantly reduced during normal S-phase of the cell cycle, which was demonstrated by DNA fiber spreading experiment. In accordance with this finding, *ING2* was also shown to interact with proliferating cell nuclear antigen, regulating its amount to the chromatin fraction, which allows for normal cell replication and cell proliferation. A high proportion of siRNA *ING2* cells showed endo-reduplication of their genome and an increased ratio of sister chromatin exchange. The authors of this study thus propose that *ING2* exerts its tumor-suppressive function by directly maintaining DNA integrity [30].

## 3. *ING3* and Its Potential Role in Lung Cancer

To the best of our knowledge, no data on the impact of *ING3* in lung cancer have been published yet. According to a review article from 2017, high expression levels of *ING3* are found in healthy tissues that feature a rapid cell proliferation, such as the small intestine, bone marrow, or the epidermis [31,32]. It was also shown that *ING3* is expressed to a significantly higher degree in proliferating versus quiescent epithelial cells. Contrary to pre-existing data on the other *ING gene* family members, data on *ING3* suggests rather pro-carcinogenic effects of this particular gene, since high levels of *ING3* correlate with more rapid cell growth. On the other hand, down-regulation of *ING3* was observed in hepatocellular carcinoma and in colorectal adenocarcinoma [33,34]. In accordance with that, loss of *ING3* was found to be associated with head and neck carcinogenesis [35]. *ING3* induces germ cell apoptosis and embryonic death in a *p53*-dependent manner [36]. The link to tumor protein p53 has also been described for *ING1b* [21]. The function of the NuA4 histone acetyltransferase multi-subunit complex is linked *p53* as well and is also mediated by *ING3* [37]. NuA4 is essential for cell cycle proliferation and DNA-repair. Thus, *ING3* is likely to impact cell proliferation by regulating NuA4. Similarly to *ING4*, interaction with *p21* and *Bax* was demonstrated for *ING3* as well: it was shown that *ING3* induced the expression of *p21* and *Bax* which resulted in apoptosis and diminished cell proliferation as shown in the RKO colon carcinoma cell line [38]. Data specifically on the role of *ING3* in lung cancer is still very limited. Since previous research has shown controversial results, i.e., *ING3* having anti-proliferative effects, whilst enhancing proliferation in other studies, it would be interesting to outline its mechanism of action in lung cancer in the future.

The ING3 protein was investigated in the context of cell cycle arrest, p53-transactivated promoters of p21 and Bcl2-associated X protein [39]. Immunohistochemistry was used to characterize ING3 expression profiles in tissue microarrays comprising various cancer specimens. Amongst these were 192 samples of lung cancer. Additionally, mouse tissue was analyzed, and ING3 cytoplasmic, as well as nuclear expression, was detected in the murine healthy bronchial and alveolar epithelial cells. Similar to ING2 [20], also ING3 was expressed in some lung cancer samples but occurred most abundantly in gynecological cancer entities. The authors conclude ING3 being involved in the repair and regeneration of tissues and point out a particular role in gynecological carcinogenesis [39].

## 4. *ING4* Down-Regulation Is Associated with an Adverse Prognosis in Lung Cancer

According to a study by Wang and colleagues from 2010, *ING4* down-regulation is closely linked to the initiation and progression of human lung cancer [40]. In this analysis, 246 lung tumors were evaluated, among them 99 samples of SCC, 129 samples of AC, three large cell lung carcinomas, four carcinoid tumors, six adenosquamous carcinomas, one unclassified lung tumor sample, and four small cell carcinomas. Expression of the ING4 protein was evaluated by means of immunohistochemistry. Nuclear as well as cytoplasmic expression levels were assessed and correlated with tumor grade. It was found that ING4 was expressed in both the nucleus and the cytoplasm of lung tumors [40]. In comparison to the healthy control tissues, most tumors featured higher cytoplasmic ING4 expression, while in the normal tissue samples, nuclear expression tended to be higher. In the tumors, nuclear expression intensity correlated with tumor stage and the presence of lymph node metastasis. To confirm the immunohistochemical findings, the authors also performed semiquantitative RT-PCR and Western blotting, both demonstrating decreased levels of *ING4* mRNA in the tumor tissues as compared to the healthy control samples. In accordance, *ING4* expression was found to be lower in grade III than in grade I or grade II tumors, and reduced *ING4* mRNA expression correlated with lymph node metastasis as well [40]. As a conclusion, *ING4* acts as a tumor suppressor in lung cancer, and its inhibition leads to initiation and progression of the disease. In the future, *ING4* might serve as a prognostic biomarker in several lung cancer subtypes [40]. In another study, similar effects of the *ING4* gene were observed in NSCLC [41]. In this analysis, an ING4/interleukin-24 (IL-24) bicistronic adenovirus (Ad-ING-IL-24) was constructed, since IL-24 has been proven to be a potent cytokine-tumor suppressor with broad-spectrum anticarcinogenic properties as well [42]. The purpose of this study was to analyze whether *ING4* and IL-24 exhibit combined anti-tumoral effects in NSCLC [41]. The effects of Ad-ING4-IL-24 transfection was investigated by means of A549 human NSCLC cell lines in vitro, as well as in vivo in a model of athymic mice who underwent subcutaneous injection of these cell lines, resulting in tumor formation. Adenovirus-mediated co-expression of *ING4* and IL-24 in NSCLC cells resulted in growth suppression and apoptosis in the lung cancer cell lines in vitro. Effects were additive for *ING4* and IL-24, and in addition up-regulation of the tumor suppressors *p21, p27,* fas cell surface death receptor *(Fas), Bax,* and the apoptosis-promoting cleaved caspases-8, -9, -3, and down-regulation of the tumor-promoter Bcl-2 apoptosis regulator *(Bcl-2)* was observed [41]. In the mouse xenograft model, Ad-ING4-IL-24 treatment resulted in synergistic inhibition of A549 lung carcinoma subcutaneous tumor growth. After the tumor-bearing mice were sacrificed, Ad-ING-IL-24 treated xenograft tumors featured a reduction of CD34, as measured by immunohistochemistry [41]. In addition, microvessel density in the xenograft tumors was reduced upon treatment with the adenovirus. The enhanced antitumoral activity was furthermore associated with activation of extrinsic and intrinsic apoptotic pathways, and inhibition of tumor angiogenesis. This study is an example of successful cancer gene therapy combining two potent tumor suppressors, which may hopefully become a therapeutic strategy for lung cancer patients in the near future.

Li and colleagues have conducted a cell culture study examining A549 lung adenocarcinoma cells with respect to *ING4* [43]. Special emphasis was put on the link between *ING4* and apoptosis. For that purpose, *ING4* cDNA was transduced into the lung adenocarcinoma cells, and FCM analysis, TUNEL assay and electron microscopy were performed. To further confirm the results, apoptosis-related proteins were analyzed by Western blotting. Both the Annexin-V FITC analysis by FCM, as well as TUNEL assay revealed higher apoptotic activity in the A549 cells with exogenous *ING4* gene. Typical apoptotic changes in cell morphology were observed via electron microscopy in the cells expressing exogenous *ING4* as well. Bcl-2 family proteins and the main apoptotic executioners of mitochondrial pathways (Cyt-c, caspase-3, and PARP) were also observed more frequently upon *ING4* transduction in the A549 cells. This data shows *ING4* to promote apoptosis in lung cancer, acting as a tumor suppressor in NSCLC, and having an impact on the mitochondrial apoptotic pathway as well [43].

In 2012, the expression of the *ING4* gene was examined in the context of docetaxel-resistant NSCLC [44]. A docetaxel-resistant lung adenocarcinoma cell line was specifically engineered (SPC-A1/DTX). *ING4* expression was evaluated by means of gene array analysis and was found to be significantly lower as compared to previous results in other NSCLC cell lines. Treatment with docetaxel further decreased *ING4* expression levels, and accordingly, *ING4* over-expression could reverse docetaxel or paclitaxel resistance in the primarily resistant cell lines (SPC-A1/DTX and A549/Taxol). In addition, induction of apoptosis and G2/M cell cycle arrest resulted from *ING4* over-expression [44]. When the researchers induced *ING4* knockdown via small interfering (si)RNA, cells became even more resistant to docetaxel or paclitaxel. Examination of samples from NSCLC patients revealed *ING4* expression levels in tumors of non-responders to chemotherapy to be significantly lower as compared to patients with good response. All the above-mentioned results highlight the tumor-suppressive function of *ING4*, and its impact on response to chemotherapy [44]. Another investigation on *ING4* was done with respect to miRNA miR-761 in progressive and metastatic NSCLC [45]. It has already been proposed, according to previous literature, that the frequently observed loss of *ING* genes is generally a result of miRNA inhibition [10]. Expression of miR-761 was evaluated by means of quantitative RT-PCR, and transwell assays were utilized to assess the role of this specific micro RNA in cell proliferation and metastatic spread. *ING4* was targeted in order to find out whether there is a link to miR-761 activity. MiR-761 was found to be up-regulated significantly in NSCLC patients, in the blood serum as well as in tissue samples, when compared to healthy patients and paired non-cancerous tissues. Luciferase reporter assay and functional analyses showed miR-761 to directly target *ING4* [45]. Tumor-suppressive properties of *ING4* are thus reversed by the cancer-promoting miR-761. A further study on a miRNA, miR-214, was carried out with the aim to enlighten a possible connection to *ING4* gene function [46]. MiR-214 was up-regulated in lung cancer tissues as compared to adjacent non-cancerous tissue. Similarly to miR-761, it was confirmed that *ING4* is a direct target of miR-214 as well, according to this study [46]. MiR-214 acted pro-carcinogenic, its over-expression leading to an up-regulation of the HIF-1alpha cascade and VEGF up-regulation in NSCLC cell lines. These results show *ING4* to act as a tumor suppressor in NSCLC.

## 5. *ING4* Expression Mediated by Adenoviruses Represses Lung Carcinogenesis

In a study by Xie et al., transgene *ING4* expression was performed in order to investigate the effects on lung carcinoma cell growth and cell cycle [47]. A549 lung cancer cells were transfected with recombinant adenoviral vectors (Ad-green fluorescent protein (GFP), Ad-ING4-GFP, Ad-DGFP (D means excision), and Ad-ING4-DGFP). To engineer the recombinant viral vectors, the humanized *ING4* cDNA fragment was subcloned into a pAdTrack-CMV vector which expresses GFP. The GFP marker gene was then excised by means of recombinant DNA technology. The pAd-Track-CMV-DGFP vector served as a control, and the recombinant Ad-GFP and Ad-DGFP adenoviruses were used as controls as well [47]. As intended, >95% of GFP expression was found in the A549 tumor cells that had been transfected with Ad-GFP or Ad-ING4-GFP, whilst GFP was not expressed in cells transfected with Ad-DGFP or Ad-ING4-DGFP. As a next step, *ING4* expression was evaluated in the total RNA and cell lysates extracted from the transfected A549 cells by means of RT-PCR and Western blot analysis. It was found that Ad-ING4 transfection led to a suppression of A549 tumor cell growth both in vitro and in vivo [47]: Cells were cultured either in the presence or absence of Ad-ING4, and the respective effects on tumor cell growth was monitored for the duration of four days via 3-(4,5-Dimethylthiazol-2-yl)-2,5-diphenyltetrazoliumbromid (MTT) assay. Inhibition of A549 cell growth upon treatment with Ad-ING4 in a time-dependent manner was significant (*p* < 0.0001). The in vivo analysis was performed in athymic nude mice bearing A549 tumors, and 6 out of 12 mice underwent intratumoral injection with Ad-ING4. In the Ad-ING4 treated mice, tumor growth was significantly attenuated as compared to the controls (*p* < 0.0001) [47]. In the same study, the effect of *ING4* on the cell cycle was analyzed via FACS analysis in the A549 tumor cells: A significant reduction in S phase, as well as an increase in the G2/M phase, was the result of Ad-ING4 treatment as compared to PBS treatment. The authors mention that this finding, indicating inhibition of tumor cell growth via reducing the S phase and an increase in the G2/M phase, differs from previous reports, where G2/M phase arrest was described as a result of adenovirus-mediated *ING4* expression in a HepG2 liver cancer cell line [48]. Furthermore, 38.7% of A549 lung cancer cells went into apoptosis upon *ING4* transfection, as compared to the control A549 cells cultured in the absence of *ING4*, where the apoptosis rate did not exceed 2% [47]. Angiogenesis also decreased in vivo according to the immunohistochemical assessment of the mouse xenograft tumor samples when injected with Ad-ING4: Tumor blood vessel expression of the hematopoietic stem cell marker CD34 was weaker, or observed in fewer cells overall, in the *ING4* treated cells in comparison to the untreated control tumors. The density of microvessels was also significantly lower in treated tumor samples [47]. In conclusion, this study by Xie and colleagues provides a valid overview of the various anti-carcinogenic anchor points of *ING4* in lung cancer.

In 2010 Moreno et al. have analyzed the specific impact of two mutations (Y121N and N214D) in the *ING4* gene, specifically associated with cancer progression in humans [49]. N214D mutations dramatically lowered the ability of *ING4* to act as a tumor suppressor, i.e., inhibition of tumor cell proliferation, cell growth and migration, and sensitization to cell death. On the contrary, the Y121N mutation did not significantly differ from wild-type *ING4* [49]. This data demonstrates a point mutation of *ING4* which is linked to human tumorigenesis, leading to the loss of several tumor-suppressive functions of *ING4*.

## 6. *ING5* Prevents Epithelial-To-Mesenchymal-Transition (EMT) and Is a Prognostic Biomarker in Lung Cancer

Zhang and colleagues have recently investigated in-depth the role of *ING5* in lung carcinoma [50]. Aberrant *ING5* expression has been demonstrated in several cancer subtypes, for example, down-regulation of *ING5* mRNA was found in squamous cell carcinoma of the oral cavity, and tumor-suppressive function of *ING5* has also been found in acute myeloid leukemia (AML) [50,51]. Furthermore, *ING5* contributes to cancer formation and disease progression in gastric carcinomas and colorectal cancer, according to previous literature [52,53]. In a recent analysis, insight into the mechanism of action of *ING5* in lung cancer is provided, with special emphasis on epithelial-to-mesenchymal-transition (EMT) which is a crucial step for tumor metastasis [50]. The authors established A549 and H1299 lung cancer cell lines stably expressing *ING5* mRNA. *ING5* mRNA and protein levels were found to be differentially expressed in human lung cancer cell lines as compared to cell lines of healthy bronchial epithelial cells: Interestingly, in the 95D and H1299 cells, *ING5* was expressed to a higher degree than in the normal bronchial epithelial cells, and in A549 and H1650 cells expression was lower [50]. In the *ING5*-over-expressing and in the *ING5*-knockdown cell lines, the effects on cell growth and invasion were investigated: It was found that *ING5* over-expression was linked to decreased cell proliferation and decreased colony formation as well, highlighting its tumor-suppressive function. Correspondingly, *ING5*-knockdown enhanced both proliferation and colony formation. Tumor cell migration was inhibited in A549 cells as shown by means of a wound healing, and a transwell migration assay, when *ING5* was over-expressed. Accordingly, the knockdown of *ING5* promoted cell migration as demonstrated in H1299 cell lines [50]. In this study, mouse xenograft models were also established to further elucidate the effect of *ING5* on tumor growth and invasion. Tumors were implanted in the mice by subcutaneous injection of A549-ING5 expressing, and A549 control cells. Tumor volumes were significantly diminished in the case of *ING5* over-expression. Furthermore, the effect of *ING5* on the process of metastasis was assessed by intravenous injection of A549-*ING5* versus A549 control cells into the tail vein of mice [50]. Forty-five days after injection, all mice (five out of five) that had been injected with the control cells developed multiple tumors in both lungs, whereas two out of five mice that were injected with A549-*ING5* cells featured lung tumors, and much fewer tumor nodules as compared to the control mice [50].

EMT as a crucial event in metastatic spread [54,55,56] was a major part of the study by Zhang et al. as well, with respect to *ING5*. Therefore, mRNA levels of several markers of EMT were evaluated in *ING*-over-expressing and in control cell lines, respectively. In the *ING5* over-expressing cells, mRNA levels of E-cadherin was elevated, whereas mRNA levels of *N-cadherin*, *SNAIL,* and *Slug* (both known as EMT transcription factors) [57] were decreased [50]. The observed changes in EMT markers were confirmed via Western blotting as well. Interestingly, a correlation with tumor cell morphology and expression of EMT markers was found: *ING5*-overexpressing A549 cells featured more cobblestone-like morphology and enhanced cluster formation as it is typical rather for epithelial cells, compared to A549 control cells that rather featured a mesenchymal phenotype with reduced cell-cell contact. These results favor the hypothesis that *ING5* acts as an inhibitor of EMT [50].

In a study from 2019 *ING5* was found to inhibit lung cancer invasion and EMT via the WNT/beta-catenin pathway [58]. The authors investigated lung cancer cell lines A549 and H1299. Western blot analysis was performed for detection of total as well as phosphorylated levels of beta-catenin and EMT-related proteins. Immunofluorescent staining was used to observe E-cadherin expression as a marker of EMT. Additionally, cell proliferation, colony formation, wound healing and migration and invasion in transwell assays were carried out to study the invasive properties of lung cancer cells. According to this investigation, *ING5* over-expression led to enhanced phosphorylation of beta-catenin at Ser33/37, leading to a decreased beta-catenin protein level [58]. In accordance, the knockdown of *ING5* mediated by small hairpin (sh)RNA significantly increased the beta-catenin level and blocked beta-catenin S33/37 phosphorylation. As a next step, cells were treated with the WNT/beta-catenin inhibitor XAV939. Treatment resulted in an inhibition of *ING5*-knockdown mediated proliferation, colony formation, migration and invasion in the lung cancer A549 cell lines. Simultaneously, an increased phosphorylation of beta-catenin S33/37 and decreased beta-catenin levels were observed. XAV939 impaired *ING5*-knockdown-induced EMT as well, which was indicated by an up-regulation of EMT markers, and decreased expression of mesenchymal markers. The WNT/beta-catenin inhibitor XAV939 was also able to block both IL-6/STAT3 pathway, as well as the PI3K/Akt signaling pathway [58]. The conclusion drawn from this study was an evident inhibition of EMT and lung cancer invasion via blocking the WNT/beta-catenin pathway by *ING5.* Another report showed *ING5* to exert tumor-suppressive properties, its knockdown in A549 cells leading to an up-regulation of the EGFR/PI3K/Akt and IL-6/STAT3 oncogenic pathways [59]. When cells were treated with niclosamide, *ING5*-knockdown induced cell proliferation was reversed, and also colony formation, migration, and invasion of the NSCLC A549 cells were inhibited. Moreover, in mouse xenograft models, niclosamide was found to impair *ING5* knockdown-stimulated cancer cell metastasis as well. EMT was also enhanced upon *ING5* knockdown [59]. Taken together, this study shows clear anti-carcinogenic properties of *ING5*, which may render it a future disease biomarker or therapeutic target.

## 7. Conclusions

Members of the *ING* gene family have anticarcinogenic properties, which holds true also for lung cancer according to the above-mentioned studies. Generally, an up-regulation of *INGs* in tumor cells results in an attenuation of cell growth and proliferation, as it was proven in cell culture experiments. Additionally, an up-regulation of EMT-associated markers was observed upon knockdown of *ING* genes, suggesting that also metastatic spread is inhibited by members of the *ING* family. Notably, there is very limited data available on the effect of *ING3* in lung cancer, whereas the function of *ING1*, *ING2*, *ING4,* and *ING5* has been investigated in lung cancer in more detail. Further research is warranted to outline whether *INGs* may be used as future prognostic or predictive biomarkers in NSCLC or SCLC in clinical routine.

## Supplementary Materials

The following are available online at https://www.mdpi.com/2072-6694/11/8/1118/s1, Table S1: *ING* genes in non-small cell lung cancer—summary of recent findings.
