# Supplementary material for: The Biological and Clinical Relevance of Inhibitor of Growth (ING) Genes in Non-Small Cell Lung Cancer
Source: Cancers (Basel). 2019 Aug 6;11(8):1118. doi: 10.3390/cancers11081118 (PMC6721451; doi:10.3390/cancers11081118)
Supplement: Supplementary file 1 [file cancers-11-01118-s001.zip › cancers-545771-SI.pptx]

## Slide 1
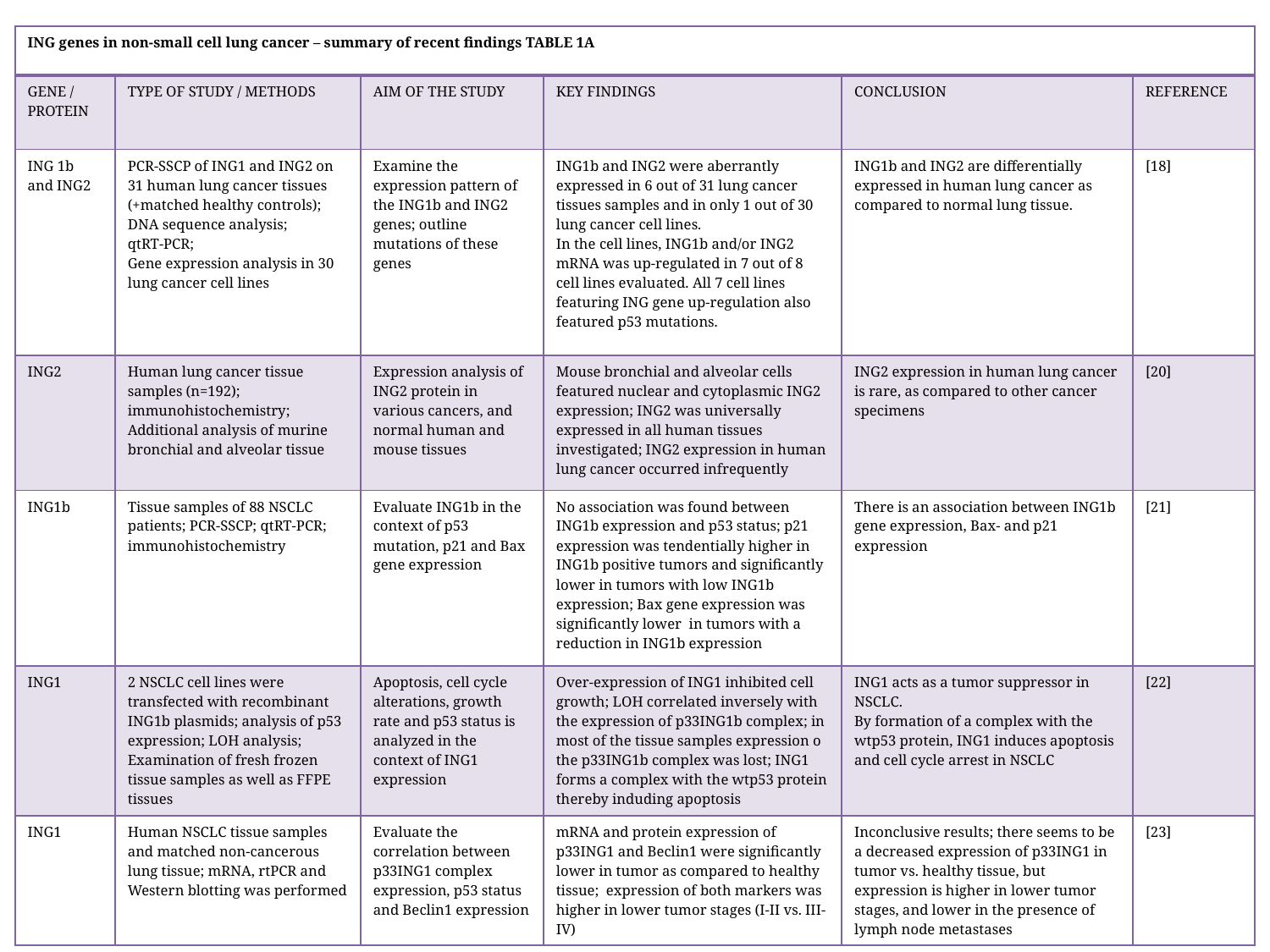

| ING genes in non-small cell lung cancer – summary of recent findings TABLE 1A | | | | | |
| --- | --- | --- | --- | --- | --- |
| GENE / PROTEIN | TYPE OF STUDY / METHODS | AIM OF THE STUDY | KEY FINDINGS | CONCLUSION | REFERENCE |
| ING 1b and ING2 | PCR-SSCP of ING1 and ING2 on 31 human lung cancer tissues (+matched healthy controls); DNA sequence analysis; qtRT-PCR; Gene expression analysis in 30 lung cancer cell lines | Examine the expression pattern of the ING1b and ING2 genes; outline mutations of these genes | ING1b and ING2 were aberrantly expressed in 6 out of 31 lung cancer tissues samples and in only 1 out of 30 lung cancer cell lines. In the cell lines, ING1b and/or ING2 mRNA was up-regulated in 7 out of 8 cell lines evaluated. All 7 cell lines featuring ING gene up-regulation also featured p53 mutations. | ING1b and ING2 are differentially expressed in human lung cancer as compared to normal lung tissue. | [18] |
| ING2 | Human lung cancer tissue samples (n=192); immunohistochemistry; Additional analysis of murine bronchial and alveolar tissue | Expression analysis of ING2 protein in various cancers, and normal human and mouse tissues | Mouse bronchial and alveolar cells featured nuclear and cytoplasmic ING2 expression; ING2 was universally expressed in all human tissues investigated; ING2 expression in human lung cancer occurred infrequently | ING2 expression in human lung cancer is rare, as compared to other cancer specimens | [20] |
| ING1b | Tissue samples of 88 NSCLC patients; PCR-SSCP; qtRT-PCR; immunohistochemistry | Evaluate ING1b in the context of p53 mutation, p21 and Bax gene expression | No association was found between ING1b expression and p53 status; p21 expression was tendentially higher in ING1b positive tumors and significantly lower in tumors with low ING1b expression; Bax gene expression was significantly lower in tumors with a reduction in ING1b expression | There is an association between ING1b gene expression, Bax- and p21 expression | [21] |
| ING1 | 2 NSCLC cell lines were transfected with recombinant ING1b plasmids; analysis of p53 expression; LOH analysis; Examination of fresh frozen tissue samples as well as FFPE tissues | Apoptosis, cell cycle alterations, growth rate and p53 status is analyzed in the context of ING1 expression | Over-expression of ING1 inhibited cell growth; LOH correlated inversely with the expression of p33ING1b complex; in most of the tissue samples expression o the p33ING1b complex was lost; ING1 forms a complex with the wtp53 protein thereby induding apoptosis | ING1 acts as a tumor suppressor in NSCLC. By formation of a complex with the wtp53 protein, ING1 induces apoptosis and cell cycle arrest in NSCLC | [22] |
| ING1 | Human NSCLC tissue samples and matched non-cancerous lung tissue; mRNA, rtPCR and Western blotting was performed | Evaluate the correlation between p33ING1 complex expression, p53 status and Beclin1 expression | mRNA and protein expression of p33ING1 and Beclin1 were significantly lower in tumor as compared to healthy tissue; expression of both markers was higher in lower tumor stages (I-II vs. III-IV) | Inconclusive results; there seems to be a decreased expression of p33ING1 in tumor vs. healthy tissue, but expression is higher in lower tumor stages, and lower in the presence of lymph node metastases | [23] |

## Slide 2
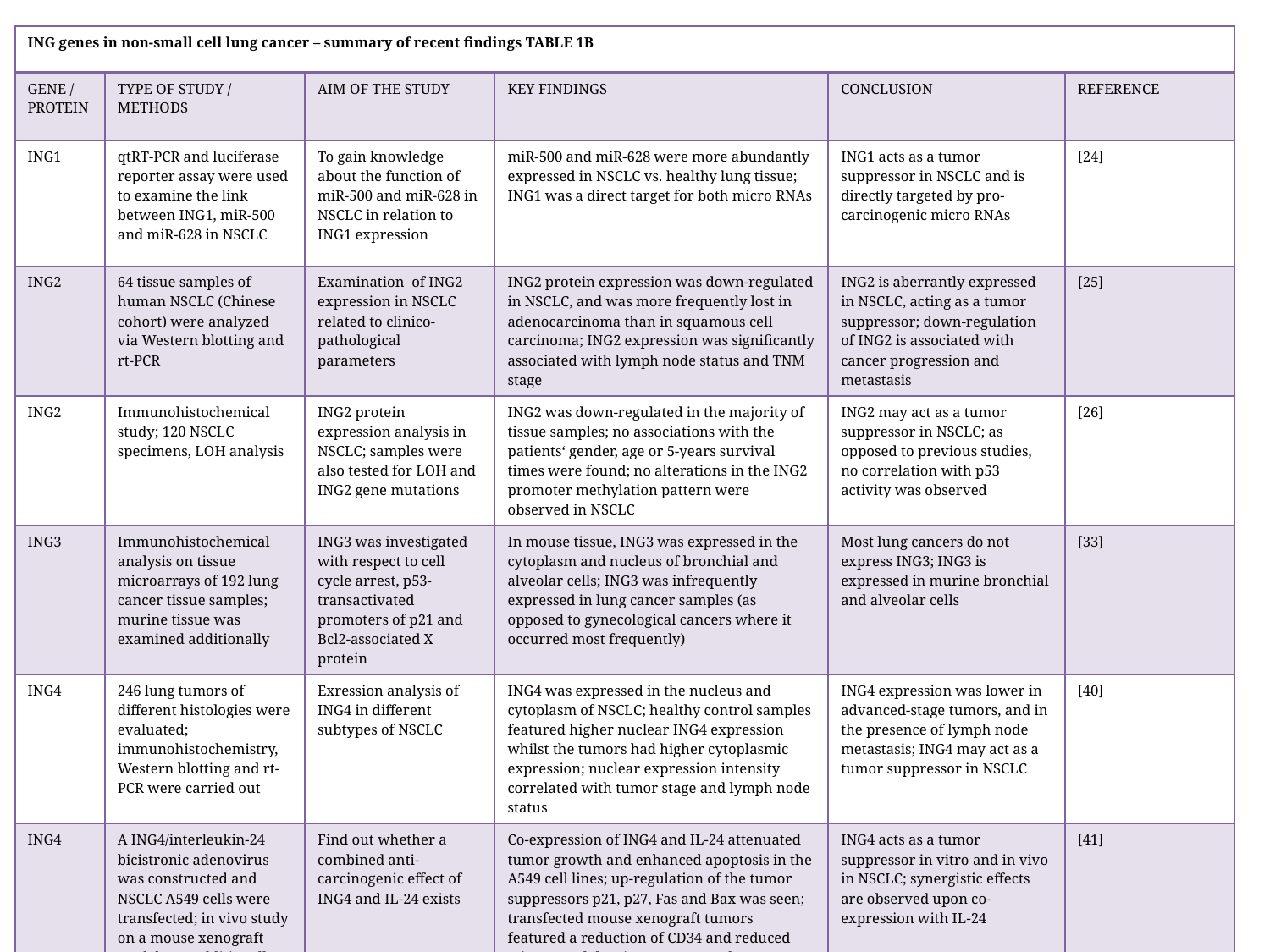

| ING genes in non-small cell lung cancer – summary of recent findings TABLE 1B | | | | | |
| --- | --- | --- | --- | --- | --- |
| GENE / PROTEIN | TYPE OF STUDY / METHODS | AIM OF THE STUDY | KEY FINDINGS | CONCLUSION | REFERENCE |
| ING1 | qtRT-PCR and luciferase reporter assay were used to examine the link between ING1, miR-500 and miR-628 in NSCLC | To gain knowledge about the function of miR-500 and miR-628 in NSCLC in relation to ING1 expression | miR-500 and miR-628 were more abundantly expressed in NSCLC vs. healthy lung tissue; ING1 was a direct target for both micro RNAs | ING1 acts as a tumor suppressor in NSCLC and is directly targeted by pro-carcinogenic micro RNAs | [24] |
| ING2 | 64 tissue samples of human NSCLC (Chinese cohort) were analyzed via Western blotting and rt-PCR | Examination of ING2 expression in NSCLC related to clinico-pathological parameters | ING2 protein expression was down-regulated in NSCLC, and was more frequently lost in adenocarcinoma than in squamous cell carcinoma; ING2 expression was significantly associated with lymph node status and TNM stage | ING2 is aberrantly expressed in NSCLC, acting as a tumor suppressor; down-regulation of ING2 is associated with cancer progression and metastasis | [25] |
| ING2 | Immunohistochemical study; 120 NSCLC specimens, LOH analysis | ING2 protein expression analysis in NSCLC; samples were also tested for LOH and ING2 gene mutations | ING2 was down-regulated in the majority of tissue samples; no associations with the patients‘ gender, age or 5-years survival times were found; no alterations in the ING2 promoter methylation pattern were observed in NSCLC | ING2 may act as a tumor suppressor in NSCLC; as opposed to previous studies, no correlation with p53 activity was observed | [26] |
| ING3 | Immunohistochemical analysis on tissue microarrays of 192 lung cancer tissue samples; murine tissue was examined additionally | ING3 was investigated with respect to cell cycle arrest, p53-transactivated promoters of p21 and Bcl2-associated X protein | In mouse tissue, ING3 was expressed in the cytoplasm and nucleus of bronchial and alveolar cells; ING3 was infrequently expressed in lung cancer samples (as opposed to gynecological cancers where it occurred most frequently) | Most lung cancers do not express ING3; ING3 is expressed in murine bronchial and alveolar cells | [33] |
| ING4 | 246 lung tumors of different histologies were evaluated; immunohistochemistry, Western blotting and rt-PCR were carried out | Exression analysis of ING4 in different subtypes of NSCLC | ING4 was expressed in the nucleus and cytoplasm of NSCLC; healthy control samples featured higher nuclear ING4 expression whilst the tumors had higher cytoplasmic expression; nuclear expression intensity correlated with tumor stage and lymph node status | ING4 expression was lower in advanced-stage tumors, and in the presence of lymph node metastasis; ING4 may act as a tumor suppressor in NSCLC | [40] |
| ING4 | A ING4/interleukin-24 bicistronic adenovirus was constructed and NSCLC A549 cells were transfected; in vivo study on a mouse xenograft model was additionally performed | Find out whether a combined anti-carcinogenic effect of ING4 and IL-24 exists | Co-expression of ING4 and IL-24 attenuated tumor growth and enhanced apoptosis in the A549 cell lines; up-regulation of the tumor suppressors p21, p27, Fas and Bax was seen; transfected mouse xenograft tumors featured a reduction of CD34 and reduced microvessel density, as compared to non-transfected controls | ING4 acts as a tumor suppressor in vitro and in vivo in NSCLC; synergistic effects are observed upon co-expression with IL-24 | [41] |

## Slide 3
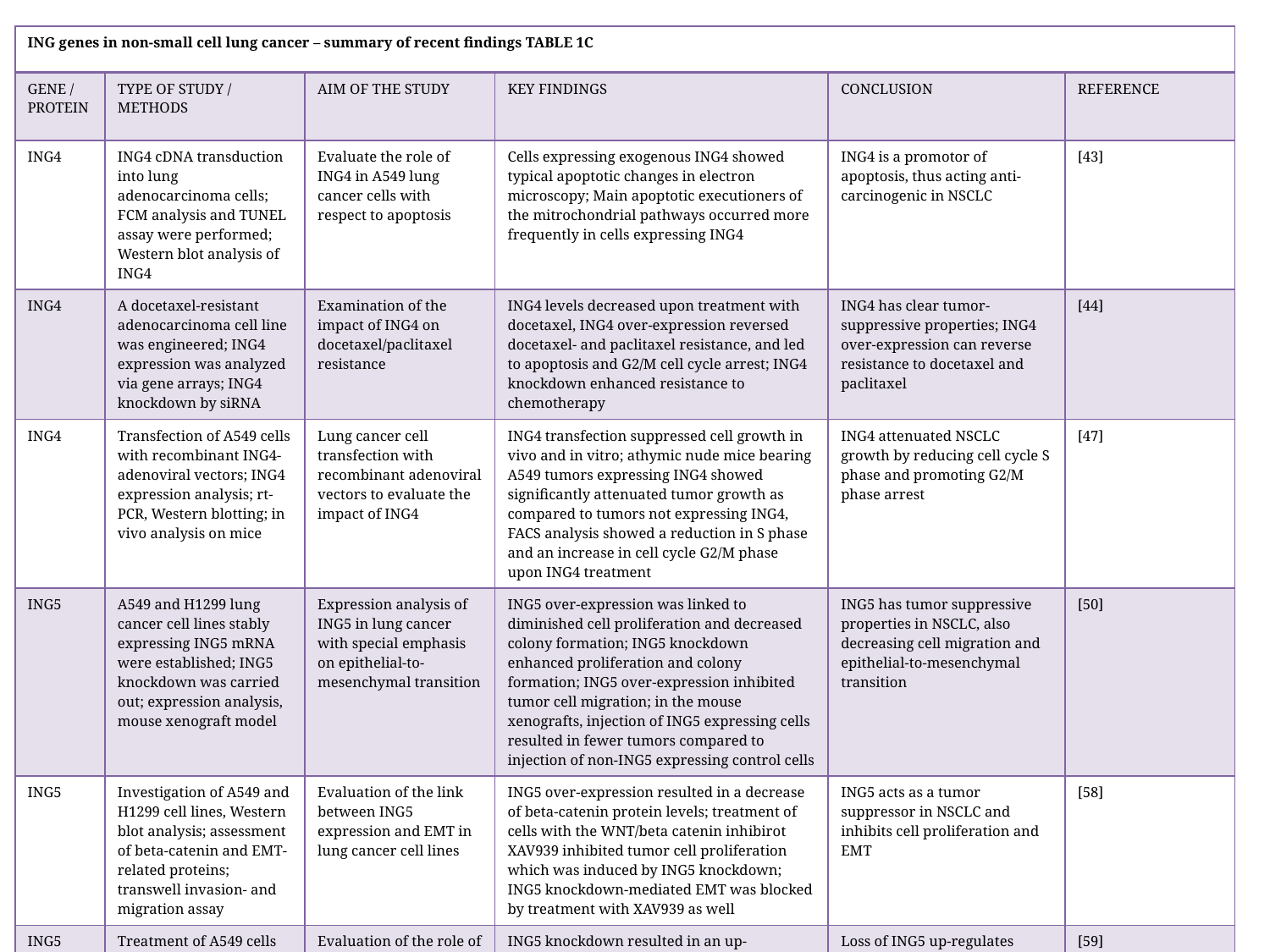

| ING genes in non-small cell lung cancer – summary of recent findings TABLE 1C | | | | | |
| --- | --- | --- | --- | --- | --- |
| GENE / PROTEIN | TYPE OF STUDY / METHODS | AIM OF THE STUDY | KEY FINDINGS | CONCLUSION | REFERENCE |
| ING4 | ING4 cDNA transduction into lung adenocarcinoma cells; FCM analysis and TUNEL assay were performed; Western blot analysis of ING4 | Evaluate the role of ING4 in A549 lung cancer cells with respect to apoptosis | Cells expressing exogenous ING4 showed typical apoptotic changes in electron microscopy; Main apoptotic executioners of the mitrochondrial pathways occurred more frequently in cells expressing ING4 | ING4 is a promotor of apoptosis, thus acting anti-carcinogenic in NSCLC | [43] |
| ING4 | A docetaxel-resistant adenocarcinoma cell line was engineered; ING4 expression was analyzed via gene arrays; ING4 knockdown by siRNA | Examination of the impact of ING4 on docetaxel/paclitaxel resistance | ING4 levels decreased upon treatment with docetaxel, ING4 over-expression reversed docetaxel- and paclitaxel resistance, and led to apoptosis and G2/M cell cycle arrest; ING4 knockdown enhanced resistance to chemotherapy | ING4 has clear tumor-suppressive properties; ING4 over-expression can reverse resistance to docetaxel and paclitaxel | [44] |
| ING4 | Transfection of A549 cells with recombinant ING4-adenoviral vectors; ING4 expression analysis; rt-PCR, Western blotting; in vivo analysis on mice | Lung cancer cell transfection with recombinant adenoviral vectors to evaluate the impact of ING4 | ING4 transfection suppressed cell growth in vivo and in vitro; athymic nude mice bearing A549 tumors expressing ING4 showed significantly attenuated tumor growth as compared to tumors not expressing ING4, FACS analysis showed a reduction in S phase and an increase in cell cycle G2/M phase upon ING4 treatment | ING4 attenuated NSCLC growth by reducing cell cycle S phase and promoting G2/M phase arrest | [47] |
| ING5 | A549 and H1299 lung cancer cell lines stably expressing ING5 mRNA were established; ING5 knockdown was carried out; expression analysis, mouse xenograft model | Expression analysis of ING5 in lung cancer with special emphasis on epithelial-to-mesenchymal transition | ING5 over-expression was linked to diminished cell proliferation and decreased colony formation; ING5 knockdown enhanced proliferation and colony formation; ING5 over-expression inhibited tumor cell migration; in the mouse xenografts, injection of ING5 expressing cells resulted in fewer tumors compared to injection of non-ING5 expressing control cells | ING5 has tumor suppressive properties in NSCLC, also decreasing cell migration and epithelial-to-mesenchymal transition | [50] |
| ING5 | Investigation of A549 and H1299 cell lines, Western blot analysis; assessment of beta-catenin and EMT-related proteins; transwell invasion- and migration assay | Evaluation of the link between ING5 expression and EMT in lung cancer cell lines | ING5 over-expression resulted in a decrease of beta-catenin protein levels; treatment of cells with the WNT/beta catenin inhibirot XAV939 inhibited tumor cell proliferation which was induced by ING5 knockdown; ING5 knockdown-mediated EMT was blocked by treatment with XAV939 as well | ING5 acts as a tumor suppressor in NSCLC and inhibits cell proliferation and EMT | [58] |
| ING5 | Treatment of A549 cells with niclosamide; ING5 expression analysis | Evaluation of the role of ING5 in NSCLC, upon treatment with niclosamide | ING5 knockdown resulted in an up-regulation of the EGFR/PI3K/Akt pathway and of the oncogenic IL-6/STAT3 pathway; ING5-knockdown-induced cell proliferation was inhibited by niclosamide | Loss of ING5 up-regulates oncogenic pathways; this effect is reversed upon niclosamide treatment | [59] |
